# Pre‐conditions for eliminating mitochondrial dysfunction and maintaining liver function after hepatic ischaemia reperfusion

**DOI:** 10.1111/jcmm.13129

**Published:** 2017-03-16

**Authors:** Chenxia Hu, Lanjuan Li

**Affiliations:** ^1^ State Key Laboratory for Diagnosis and Treatment of Infectious Diseases Collaborative Innovation Center for Diagnosis and Treatment of Infectious Diseases First Affiliated Hospital School of Medicine Zhejiang University Hangzhou Zhejiang China

**Keywords:** hepatic, ischaemia reperfusion, liver transplantation, pre‐condition, mitochondria

## Abstract

The liver, the largest organ with multiple synthesis and secretion functions in mammals, consists of hepatocytes and Kupffer, stem, endothelial, stellate and other parenchymal cells. Because of early and extensive contact with the external environment, hepatic ischaemia reperfusion (IR) may result in mitochondrial dysfunction, autophagy and apoptosis of cells and tissues under various pathological conditions. Because the liver requires a high oxygen supply to maintain normal detoxification and synthesis functions, it is extremely susceptible to ischaemia and subsequent reperfusion with blood. Consequently, hepatic IR leads to acute or chronic liver failure and significantly increases the total rate of morbidity and mortality through multiple regulatory mechanisms. An increasing number of studies indicate that mitochondrial structure and function are impaired after hepatic IR, but that the health of liver tissues or liver grafts can be effectively rescued by attenuation of mitochondrial dysfunction. In this review, we mainly focus on the subsequent therapeutic interventions related to the conservation of mitochondrial function involved in mitigating hepatic IR injury and the potential mechanisms of protection. Because mitochondria are abundant in liver tissue, clarification of the regulatory mechanisms between mitochondrial dysfunction and hepatic IR should shed light on clinical therapies for alleviating hepatic IR‐induced injury.

## Introduction

The liver, the largest organ with multiple synthesis and secretion functions in mammals, consists of hepatocytes and Kupffer, stem, endothelial, stellate and other parenchymal cells. Because of the early and extensive contact with the external environment, hepatic ischaemia reperfusion (IR) may result in mitochondrial dysfunction, autophagy and apoptosis of cells and tissues under various pathological conditions. Because the liver requires a high oxygen supply to maintain normal detoxification and synthesis functions, it is extremely susceptible to ischaemia and subsequent reperfusion with blood 1. Hepatic IR, a frequent clinical outcome of pathological conditions, may result in mitochondrial dysfunction, a wave of parenchymal cell death and an abundance of cytotoxic inflammatory responses 2. Consequently, hepatic IR leads to acute or chronic liver failure and significantly increases the total rate of morbidity and mortality through multiple regulatory mechanisms.

Under different pathological conditions, hepatic IR injury can be classified into different types according to the environmental temperature. Warm IR injury has been observed in low‐flow situations 3, 4; in contrast, cold IR injury has been observed solely in liver transplantation (LT) in which the excised graft was subjected to hypothermic preservation before warm reperfusion 5. In clinical conditions, warm IR occurs more frequently than cold hepatic IR and leads to initial deficiencies and incompetence of liver allografts. Furthermore, most of the animal models that have been used in experimental studies are hepatic partial hepatectomy models that imitate warm hepatic IR to induce hepatocellular damage and apoptosis. Before LT, the liver graft is preserved in cold buffer, and reperfusion induces apoptosis in sinusoidal endothelial cells because of the characteristic lack of organized basal membrane^5^. LT is the ultimate choice for end‐stage liver disease because it induces hepatic injury and apoptosis of hepatocytes and other cells, and hepatic IR leads to liver graft dysfunction.

**Figure 1:**
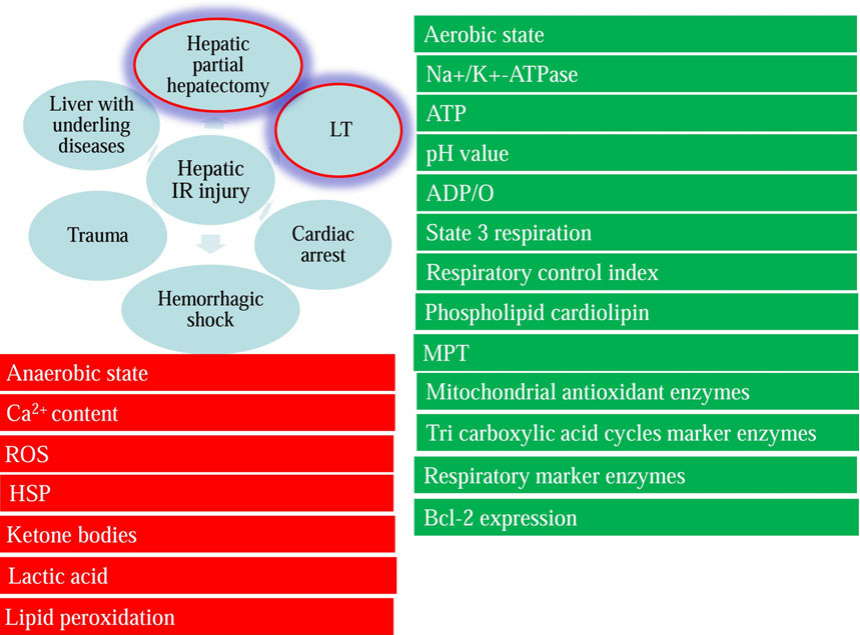
Hepatic IR can be classified into warm IR and LT‐induced IR; the parameters in the red boxes are upregulated, and the parameters in green boxes are down‐regulated during the hepatic ischaemic process.

Mitochondria play a role as the primary target in an early stage of hepatic IR (Fig. 1), and the increased level of reactive oxygen species (ROS) promotes the release of various inflammatory signals and adversely affects the metabolic processes in liver tissues 6. During hepatic ischaemia, aerobic energy metabolism is shifted to an anaerobic state, accompanied with a decrease in respiratory enzymes and mitochondrial antioxidant enzymes for adenosine triphosphate (ATP) production, but the accumulation of lipid peroxidation and Ca^2+^ content is initiated after hepatic IR 7. The attenuation of mitochondrial permeability transition (MPT), phospholipid cardiolipin, the respiratory control index, ADP/O, and state 3 respiration have also been observed during the pathological changes 8, 9. Although the synthesis of pH‐dependent enzymes is activated and the pH values are restored to primary levels after reperfusion, the impairments continue to extensively and aggressively damage the liver 10. Additionally, inflammatory cells and various cytokines are released as a defence against the adverse effects 11, and the activities of nicotinamide adenine dinucleotide NAD (NADH) cytochrome c reductase and succinate cytochrome c reductase are not consistently decreased after reperfusion. Consequently, the severity of hepatic IR injury cannot be predicted by solely the expression levels of mitochondrial respiratory enzymes 12. Moreover, the protein expression levels of 234 genes have been found to be significantly altered after hepatic IR, according to a proteomic analysis of liver mitochondria 13. Although all these changes contribute to the microcirculation failure and cellular destruction during hepatic IR, it has been demonstrated that the initial injury can be inhibited by the attenuation of mitochondrial dysfunction 14. Heme oxygenase‐1 (HO‐1) and heat shock protein (HSP)‐70 are upregulated to conserve the cytoskeleton integrity and protect against hepatic IR‐induced injury 15. The expression of HO‐1 is regulated through the inhibition of tumour necrosis factor (TNF)/TNF receptor 1, specifically by the modulation of the death‐inducing signalling complex formation and mitochondrial TNF receptor 1 translocation during hepatic IR 16. Additionally, the p38‐mitogen‐activated protein kinase (MAPK) as well as the extracellular signal‐regulated kinases 1/2 (ERK 1/2) signalling pathways eliminate hepatic IR injury 17.

**Figure 2:**
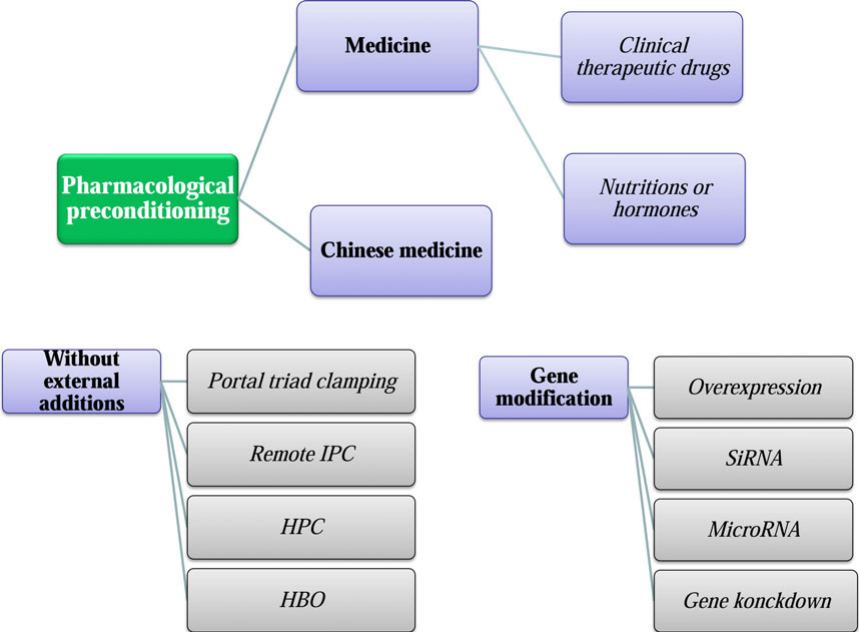
Pre‐conditions are categorized into three categories, and pharmacological pre‐conditions that have been investigated in recent years are marked in green.

Hepatic IR injury induces apoptosis and necrosis of cells and leads to irreversible damage if this process is not intervened timely. Apoptosis is an orderly form of cell death for clearance of unneeded cells to keep natural homeostasis during development, growth or ageing; in contrast, necrosis is marked by rapid loss of plasma membrane integrity and stimulated inflammation 18. In general, apoptosis rate can be determined by specific markers including Bax, caspase, PARP‐1,TNF, TUNEL, etc 19, 20, 21, but it now appears that these markers will not specifically distinguish between apoptosis and necrosis 22. In hepatic IR model, hepatic necrosis rate is closely correlated with alanine aminotransferase (ALT) levels, and this phenomenon is more apparent after 24 hr of reperfusion 23. In addition, hepatic IR model shows reduced expression levels of mitochondrial enzymes accompanied by increasing number of necrotic and apoptotic cells 24. However, apoptosis was also termed as the early mechanism while necrosis was termed as the principal mechanism of cell death in hepatic IR models 25, 26. In view of this controversy, necroapoptosis was proposed to emphasize both necrosis and apoptosis exist in hepatic IR models 27. Although necroptosis is determined as programmed necrosis *via* receptor‐interacting protein 1 pathway, it is not always present in the post‐ischaemic liver but correlated to caspase activation 28. To eliminate adverse outcomes, an increasing amount of pre‐treatments (Fig. 2) including ischaemic pre‐conditioning (IPC), pre‐treatments involving exposure to non‐physiological oxygen levels, pharmaceutical pre‐conditioning, and gene targetting approaches that can significantly reduce injury after hepatic IR are under investigation. In this review, we mainly focus on the subsequent therapeutic interventions related to the conservation of mitochondrial function involved in mitigating hepatic IR injury and the potential mechanisms for the protection. Because mitochondria are abundant in liver tissue, elucidation of the regulatory mechanisms between mitochondrial dysfunction and hepatic IR‐induced injury should shed light on clinical therapies for alleviating hepatic injury during various pathological injuries.

## IPC for attenuating hepatic IR injury

Multiple clinical and animal studies have demonstrated that IPC improves the outcomes of hepatic IR by influencing the survival rates of hepatocytes and non‐parenchymal cells 29. IPC is executed as a short period of portal triad clamping and a subsequent longer period of reperfusion, and then, the tissues or organs are incubated during a relatively long period of ischaemia 30. During the brief ischaemic exposure before hepatic IR, an enzyme with antioxidant properties, namely HO‐1, is initially activated and consequently maintains normal function 31. After clamping of the hepatic blood vessels, there is a mild increase in peroxides, which stimulates cellular adaptation 32 and mitochondrial preservation 33. This adaptive prerequisite condition notably conserves hepatic ATP synthase activity, ATP level and tolerance to MPT 34. In multiple clinical tests, it has concluded that IPC results in a decreased hospital length of stay and decreased transfusion rates 35. Although IPC seems to have a protective effect against hepatic IR injury, it may exert noxious effects on small liver remnants 36. Recently, Ye *et al*. 37 have demonstrated that IPC exerts no apparent effects on decreasing the level of serum transaminases and rates of morbidity and mortality. Because there is inevitable heterogeneity among patients, IPC varies with ischaemia time and respiration time, and an IPC protocol has various influences on hepatic IR. Additionally, compared with topical hypothermia, IPC cannot decrease inflammatory cytokines, inducible nitric oxide synthase (iNOS) and nicotinamide adenine dinucleotide phosphate (NADPH)‐quinone oxidoreductase‐1 as efficiently as topical hypothermia, and the combination of the two pre‐treatments confers no additional advantage 38.

Because IPC through portal triad clamping is complicated to execute, remote IPC through other parts of the body can be easily controlled. Lai *et al*. 39 have demonstrated that remote IPC on the limbs results in favourable changes in mitochondrial oxygenation, serum bicarbonate and hepatic venous nitric oxide (NO), NO protects against MPT‐dependent apoptosis through the cGMP‐dependent kinase signalling pathway 40. One study has executed IPC in the right hind limbs of rats with six cycles of pre‐condition clamping and reperfusion and has found that the attenuated liver necrosis is mediated by the up‐regulation of interleukin(IL)‐6 41. In addition to remote IPC on limbs, intestinal IPC has also eliminated hepatic IR‐induced injury by decreasing inflammatory cytokines and enhancing Bcl‐2 expression 42. However, investigators also found that there was no additional protective effect of IPC in patients undergoing liver resection under continuous or intermittent vascular occlusion 43. Consequently, the detailed protocols for clinical usage of IPC should be adjusted according to the detailed conditions.

## Pre‐treatments with un‐physiological oxygen content before hepatic IR

Other techniques in addition to clamping have been used to obtain an ischaemic environment; hypoxic pre‐conditioning (HPC) has been executed by exposing the liver to an altitude chamber for several days, and the release of protective protein, namely HO‐1, is significantly induced with a diminished elevation of serum ALT levels after hepatic IR 39. Hypoxic pre‐conditioning on isolated liver grafts also up‐regulates Bcl‐2 expression and significantly decreases the apoptosis rate after hepatic IR 44. One study has found that mice receiving breathing oxygen at a low concentration before hepatic IR, compared with control mice, released lower levels of apoptotic cytokines and myeloperoxidase 45.

Hyperbaric oxygen (HBO) therapy at several absolute atmospheres can improve the prognosis of severe diseases including acute brain injury, and acute lung injury. 46, 47. The protective effects of liver injury in HBO pre‐conditioning remain controversial because this pre‐treatment does not increase hepatocellular energy metabolism or repair mitochondrial oedema after hepatic IR 48, 49. Intriguingly, Losada *et al*. 50 have reported that HBO pre‐conditioning protects the liver from mitochondrial dysfunction and decreases the levels of hepatic injury markers during the ischaemia and reperfusion processes. Although HBO pre‐conditioning has been proven to effectively eliminate ALT and to improve the expression of HO‐1, it cannot decrease the levels of aspartate aminotransferase (AST) and malondialdehyde (MDA) 51, 52. Notably, the time of HBO exposure is also critical for determining whether the effects in hepatic IR models are beneficial or deleterious 53. Yu *et al*. 54 have shown that pre‐conditioning by one‐dose HBO but not three‐dose HBO effectively inhibits subsequent hepatic IR injury in rats. The pre‐condition of oxygen concentration should be further compared to optimize the protection of liver mitochondrial function without additional pharmacokinetics.

## Pharmacological pre‐conditioning before hepatic IR

Various experiments have demonstrated that pharmacological pre‐conditioning may protect liver tissue against IR injury and maintain the mitochondrial function or may alter the signalling pathways attenuating the functional impairments. Pharmacological treatments for preventing hepatic IR injury typically have damaging side‐effects in patients, and it is crucial to develop new drugs that are safe and usable for clinical tests or applications. Moreover, because of the lack of adequate clinical trials, pharmacological pre‐conditioning for reducing hepatic IR‐induced injury remains a controversial issue.

### Clinical therapeutic drugs

Many therapeutic drugs have been tested and demonstrated to have protective effects in mitochondrial function after hepatic IR injury (Table 1).

**Table 1:** Clinical therapeutic drugs and their potential mechanisms for hepatic warm IR

Piperazine	Potential mechanisms	Model	References
Cyclosporine	Preventing MPT and decreasing cytochrome c release	Mouse	58
Minocycline/ doxycycline	Inhibiting mitochondrial Ca^2+^ uptake and eliminating the Ca^2+^‐induced MPT	*In vitro* rat hepatocytes	55
Carbamazepine	Preventing calcium overload and calpain activation	Mouse	56
17β‐estradiol	Decreasing the apoptosis rate of hepatocytes by up‐regulating the ratio of Bcl‐2/Bax, decreasing cytochrome c release, and decreasing activities of caspase‐related genes, consequently improving the 7‐day survival rate	Rat	85
Gadolinium chloride	Inhibiting the release of serum aminotransferases and TNF‐α, decreasing mitochondrial MDA and suppressing the release of caspase‐3	Rat	62
Thrombomodulin	Protecting against hepatectomy‐induced macrophage/monocyte infiltration and improving the proliferation rate of hepatocytes	Rat	65
Amlodipine	Prohibiting the uptake of mitochondrial Ca^2+^ and inhibiting the Ca^2+^‐induced MPT	Rat	74
Edaravone	Suppressing the IR‐induced disorganization of mitochondrial structures	Rat	138
Levosimendan	Enhancing the hepatic microcirculation and decreasing histological damage, serum aminotransferase level, DNA damage and liver redox homeostasis	Rat	75 76
Diazoxide	Decreasing liver mitochondrial dysfunction, but the MDA content and MPO activity were not affected	Rat	77
Vinpocetine	Inhibiting the release of IL‐1β and IL‐6 while enhancing the expression of GSH	Rat	68
CV159	Decreasing the release of HMGB‐1 and iNOS but elevating the level of eNOS	Rat	59
Eritoran	Preventing inflammatory cellular responses by inhibiting HMGB1‐mediated inflammatory signalling	Mouse	60
Remifentanil	Improving MMP and inhibiting mitochondrial swelling and synthesis of superoxide dismutase, simultaneously decreasing high levels of IR‐induced TNF‐α and NF‐κB‐p65	Rat	70
Propofol	Preserving the respiratory activity and normal energy metabolism, thus limiting free radical production and PTP opening promoting the phosphorylation of mitochondrial GSK‐3β at Ser9, and consequently restraining the opening of MPT and MMP collapse	Rat	71 72
Flurbiprofen	Preserving respiratory activity and normal energy metabolism, thus limiting free radical production and PTP opening, promoting the phosphorylation of mitochondrial GSK‐3β at Ser9, and consequently restraining the opening of MPT and MMP collapse	Mouse	139

Some drugs that directly interfere with mitochondrial metabolism are widely used in current therapies. Pre‐treatment with minocycline and doxycycline decreases the uptake of mitochondrial Ca^2+^ and eliminates the Ca^2+^‐mediated MPT 55; additionally, carbamazepine prevents calcium overload and calpain activation, which are induced by IR 56. CF102, as an effective A3 adenosine receptor agonist, promotes the proliferation of hepatocytes and rescues damaged liver function by down‐regulating the nuclear factor kappa B (NF‐κB) signalling pathway 57. *N*‐methyl‐4‐isoleucine cyclosporine improves liver regeneration after massive hepatectomy by preventing MPT and decreasing cytochrome c release 58. The high‐mobility group box protein B1 (HMGB1) is positively associated with significant damage and metabolic imbalance in the liver, and a calcium channel blocker named CV159, which significantly decreases the release of HMGB‐1 and iNOS but elevates the level of eNOS, can protect against hepatic IR‐induced injury 59. Eritoran, an inhibitor of toll‐like receptor (TLR)‐4, also prevents inflammatory cellular responses by inhibiting HMGB1‐mediated inflammatory signalling 60.

Recent studies have demonstrated that hepatic IR injury is closely related to acute inflammatory responses and the release of Kupffer cells, monocytes and neutrophils. IL‐18 binding protein significantly decreases the IR‐induced liver injury and necroapoptosis of Kupffer cells, whereas blocking IL‐18 inhibited the release of NF‐κB, c‐Jun, myeloperoxidase and IL‐32 and concurrently up‐regulates inflammatory neutrophils and lymphocytes 61. Gadolinium chloride, a drug inhibiting Kupffer cells, inhibits the levels of serum aminotransferases and TNF‐α, decreases mitochondrial MDA and suppresses the release of caspase‐3 after hepatic IR 62. Tripeptide glutathione (GSH), which has substantial antioxidant properties, is present in hepatocytes at high concentrations 63, and GSH detoxifies ROS generated by Kupffer cells with or without peroxidase 64. Thrombomodulin protects against hepatectomy‐induced macrophage/monocyte infiltration and significantly improves the proliferation rate of hepatocytes 65. Although cilostazol up‐regulates the expression of HO‐1, mitochondrial biogenesis and mtDNA content, the protective effects are neutralized by the inhibition of HO‐1 or nuclear factor E2‐related factor 2(Nrf2) 66. To the best of our knowledge, many antineoplastic drugs result in strong inflammatory responses *in vivo* and inhibit tumour cells from proliferating and progressing, thus leading to multiple organ failure. Interestingly, these drugs have also been found to have antitoxic effects on hepatic IR‐induced injury: infliximab significantly inhibits inferior impairments *in vivo* models, owing to its selective antioxidant and anti‐TNF‐α effects 67. Vinpocetine inhibits the release of IL‐1β and IL‐6 but enhances the expression of GSH in warm hepatic IR rat models 68. As a strong immunosuppressive agent, FK506 decreases apoptosis in sinusoidal endothelial cells and inhibits the activation of inflammation, consequently maintaining normal microcirculation in hepatic IR animals 69.

Anaesthetics or analgesics are used to eliminate the perception of pain in patients, but these drugs have also been found to decrease hepatic IR injury. Remifentanil pre‐conditioning improves mitochondrial membrane potential (MMP) and inhibits mitochondrial swelling and synthesis of superoxide dismutase while simultaneously decreasing IR‐induced high levels of TNF‐α and NF‐κB‐p65 in liver tissues 70. By inhibiting the expression of hypoxia inducible factor (HIF)‐1α, propofol preserves the respiratory activity and normal energy metabolism, thus limiting free radical production and permeability transition pore (PTP) opening 71. Furthermore, propofol promotes the phosphorylation of mitochondrial glycogen synthase kinase (GSK)‐3β at Ser9, thus consequently restraining the opening of MPT and MMP collapse 72. Sevoflurane pre‐treatment decreases the release of heparan sulphate and syndecan‐1 and attenuates the release of serum aminopherases in a time‐dependent manner, thus decreasing the shedding of endothelial glycocalyx and cell death rate 73.

Intriguingly, an increasing number of antihypertensive drugs, including angiotensin‐converting enzyme inhibitors, beta blockers, diuretics and calcium channel blockers, for eliminating the impairment from hepatic IR injury are under investigation. For example, amlodipine blocks the uptake of mitochondrial Ca^2+^ and inhibits Ca^2+^‐induced MPT 74. Pre‐treatment with levosimendan, a calcium sensitizer, significantly enhances hepatic microcirculation and decreases histological damage, the serum aminotransferase level, DNA damage and liver redox homeostasis 75. Additionally, levosimendan suppresses the release of Bax, caspase‐9, AKT and endothelial nitric oxide synthase (eNOS) in hepatic IR models; oxidative damage induced by hepatic IR injury have also been found to be attenuated in a dose‐dependent manner 76. Although diazoxide significantly decreases liver mitochondrial dysfunction after hepatic IR, the MDA content and myeloperoxidase (MPO) activity are not affected in the liver 77. A similar drug, trimetazidine, effectively rescue hepatic IR injury in rat model, but repeated administration of this drug confers more protection by significantly enhancing signalling pathways including phosphorylated adenosine monophosphate‐activated protein kinase (AMPK) and eNOS 78.

### Drugs affecting nutrition or hormone levels

In addition to the powerful drugs described above, drugs affecting nutrition or hormone levels can also affect the maintenance of mitochondrial function and hepatocellular structure. Intravenous glycine administration results in the maintenance of cellular energy production and decreases cytokine levels and hepatocellular injury by increasing the fluxion of portal blood and hepatic microcirculation, thus preserving the plasma membrane and cytochrome oxidase activity after warm hepatic IR 79, 80. Additionally, pharmaceuticals including desferal and menadione effectively inhibit mitochondrial calcein quenching and mitochondrial ROS and MPT, thus inhibiting hepatic IR injury 19, 81. Protein kinase A, a specific peptide inhibitor, effectively abolishes cytosol‐induced inhibition of MPT and hepatic IR injury 82. Owing to its high water‐solubility and stability, a novel selenocysteine containing the 7‐mer peptide attenuates hepatic IR‐induced injury by suppressing free radicals and up‐regulating Bcl2/Bax expression 83. Polyethylene glycol‐conjugated cysteine‐modified lysine dendrimers eliminate ROS production and prolong circulation time in hepatic IR injury 84. The basal levels of hormones determine the level of damage resistance *in vivo*, and it is important to maintain or enhance some hormones *in vivo* to improve the outcomes in hepatic IR models. 17β‐estradiol has been found to decrease the apoptosis rate in hepatocytes by up‐regulating the ratio of Bcl‐2/Bax, to decrease cytochrome c release, and to decrease the activities of caspase‐related genes, thereby improving the 7‐day survival rate after hepatic IR injury 85. Pre‐treatment with flavonoids, which inhibit the TLR4 pathway but activate the Sirt1/Nrf2 pathway, down‐regulate IL‐1β, IL‐6 and TNF‐α but improve oxidative stress in hepatic IR rats 86. Because of its less side‐effect, this kind of drugs may serve as a novel and useful route to repair hepatic IR‐induced injury.

### Chinese medicine

Intriguingly, multiple Chinese medicines have emerged as treatments for hepatic IR injury, although some Chinese medicines have previously been found to be toxic and can lead to hepatic dysfunction. Paeoniflorin attenuates hepatic IR injury by acting as a strong immunosuppressor 87. Trans‐resveratrol negatively alters the production of related cytokines and reverses the TLR4/NF‐κB signalling pathway in liver tissues undergoing hepatic IR 88.

Tetrandrine, a traditional Chinese medicine with calcium channel blocking capacity exerts a protective effect on hepatic IR models by reducing oxidative stress and maintaining superoxide dismutase activity 89. Astaxanthin significantly decreases hepatic production of xanthine oxidase and protein carbonyl after IR, whereas parenchymal cell damage, mitochondrial swelling, and impairment of rough endoplasmic reticulum are reversed to some extent 90. Recently, catechins, which are extracted from green tea, have been found to be protective by maintaining manganese superoxide dismutase (MnSOD) 91. Additionally, sulforaphane exerts antioxidant effects in a rat hepatic IR model by activating the Nrf2 signal, ameliorating oxidative stress, and preserving ATPase activity 92. Cryptotanshinone attenuates serum aminotransferase levels and hepatic IR injury by inhibiting the c‐Jun N‐terminal kinase (JNK) and MAPK signalling pathways 93. Eupatilin, a pharmacologically active flavone derived from the Artemisia species, promotes the accumulation of HSP and Bcl‐2, attenuates the release of iNOS and cleaves caspase‐3 after hepatic IR 94. All these Chinese medicines demonstrate protective effects *via* multiple signalling pathways including calcium resistance, immunosuppression and interference in energy metabolism.

## Genetic modification strategies for warm hepatic IR injury

In addition to external pre‐treatments involving ischaemic environments or pharmacokinetics, recent studies have proposed gene targetting strategies to decrease hepatic IR injury. The loss of Plexin C1 decreases injuries induced by hepatic IR, as evidenced by lower levels of LDH, aspartate and ALT and fewer neutrophils in ischaemic hepatic tissue 95. The inhibition of gene transcription exerts anti‐inflammatory response and anti‐apoptosis effects on these models. For example, genetic ablation of CCL2 decreases the levels of neutrophil recruitment 96, and knockdown of TNF‐α‐induced‐protein 3 inhibits Bax expression and mitochondrial apoptosis 97. Although no differences in the cell death rate have been found in JNK2‐knockout mice, these mice exhibit less ALT release and less necrosis, a higher survival rate and overall better Kaplan‐Meier survival 98. Sphingosine kinase‐2 inhibition decreases the levels of NO synthase, NFκB‐p65 and TNF‐α; thus, mitochondrial depolarization, MPT and neutrophil infiltration are down‐regulated *in vivo* and *in vitro* hepatic IR models; intriguingly, the inhibition significantly increases the survival rates of mice after hepatic IR 99.

Most gene targetting investigations have focused on the inhibitory effects of genes on hepatic IR injury, some genes play critical roles in maintaining normal liver functions. Prdx6‐knockout mice exhibit more mitochondrial dysfunction and hepatocellular injury, and the generation of mitochondrial hydrogen peroxide is increased 13. Furthermore, overexpression of Nrf2 decreases the release of inflammatory cytokines and 8‐isoprostanes, thus protecting the liver against hepatic IR injury 100. A plasmid with a receptor activator for NF‐κB‐Fc has been found to exert prominent effects, protecting against hepatocellular apoptosis in hepatic IR mice by inhibiting NF‐κB nuclear translocation, JNK phosphorylation and HIF‐1α expression 101. Since TLR is primarily recognized as a vector in immunoreactions 102, galactose‐conjugated liposome nanoparticle TLR4 siRNA delivery have been found to efficiently attenuate neutrophil and lipid peroxidase accumulation and to suppress IL‐1 and TNF‐α expression as well as hepatic IR injury in a mouse model 103. In the same manner, the single‐stranded and non‐coding small RNA microRNA‐370 targets 3′ un‐translated regions of TGF‐β receptor II and induces hepatic IR injury, and inhibition of microRNA‐370 decreases the levels of serum aminotransferase and pro‐inflammatory cytokines 104.

## Pre‐conditions for LT to eliminate cold hepatic IR injury

According to the complex processes in LT including partial or whole liver procurements, conservation in destined buffer, organ rewarming and final transplantation, isolated liver tissues cumulatively acquire injuries because of the gradual exposure to non‐physiological and harmful conditions. Unfortunately, some drugs exert side‐effects on the LT procedure and result in serious damage in liver grafts. For example, colloid hydroxyethyl starch prevents interstitial oedema when added into UW preservation solution but lead to stasis of blood and incomplete wash out of donor organs before transplantation 105, 106. On the other hand, intravenous administration of pan‐caspase inhibitors to the recipient abolished the previously observed protective effects 107. Although most of the administration during LT provided promising results, a multicentre studies are still necessary for confirming these results.

### Pre‐conditions for excised liver grafts

In addition to these impairments in LT, the conservation of function in an excised liver graft is more important than that that observed in warm IR, and the optimization of protocols can be extremely useful for improving the survival rates and prognosis.

Before the extraction of liver grafts, IPC partly alleviates operation‐induced liver injury and reduces the IL‐6 level, thereby inhibiting the generation of multiple inflammatory cytokines 108; thus, the survival time of the liver graft is prolonged 109. Additionally, the 90‐min hypoxic environment before LT notably increases the expression level of HIF‐1α and protects against hepatic IR injury by promoting glucose metabolism 110. Addition of ulinastatin and simvastatin inhibits the release of inflammatory cytokines and apoptotic genes in a dose‐dependent manner, thus allowing liver grafts to withstand cold IR 111, 112. Bortezomib efficiently protects liver grafts against cold IR injury even at low doses and is thus considered a powerful protective agent for the maintenance of liver function 113. Even in reduced‐size LT models, bortezomib has been found to decrease oxidative stress, ER stress, mitochondrial dysfunction and hepatic IR injury; the expression levels of some well‐known IR protective proteins including NO synthase, HO‐1 and HSP70 are upregulated, and liver regeneration is accelerated 114. Addition of activated protein C (APC) to the preservation solution exerts cytoprotective effects by decreasing portal pressure, inflammatory cytokine release and hepatocellular apoptosis 115. Furthermore, the combination of Institute Georges Lopez 1 with trimetazidine in a preservation solution eliminates LT‐induced injury by up‐regulating sirtuin 1, AMPK signalling and inhibiting mTOR signalling 116. As mentioned above, some protocols of genetic modification effectively improve abnormal liver function in warm IR models and also sustain hepatocellular function in LT models. Injection of a recombinant adenovirus with Bcl‐2 into the portal vein of a rat donor liver and subsequent refrigeration of the liver graft at 4°C for 4 hr has been found to significantly increase hepatic Bcl‐2 expression and decrease the LDH level 117.

### Pre‐treatments for liver recipients

After transplantation with excised liver grafts, portal blood is perfused to initiate hepatic function recovery. Although serum pH values normalize slowly after hepatic IR, the heamodynamics stabilize quickly in LT models 118. Losartan administered to donors and recipients before LT has been found to result in the maintenance of liver function by inhibition of multiple damaging signalling pathways 119. The dichloroacetate diisopropylamine pre‐treatment in liver recipients is associated with a preservation of hepatocellular mitochondria and promotes the recovery of donor liver function after LT by down‐regulating the release of cytochrome c 120. The carbonic anhydrase inhibitor acetazolamide protects against cold hepatic IR injury by decreasing the MAPK signal and up‐regulating eNOS 121. Dexmedetomidine alleviates hepatic IR injury by suppressing intercellular adhesion molecule 1 and improving post‐operative liver function 122. Additionally, Chinese medicines have also been shown to conserve liver function in LT models. For example, Sprague‐Dawley rats that administered astragaloside IV before surgery have higher survival rates as a result of down‐regulation of TNF‐α levels and NF‐κB expression 123.

The JNK pathway is upregulated after LT, and knockout of JNK2 decreases lipid peroxidation, mitochondrial cytochrome c release, mitochondrial depolarization and hepatocellular necrosis 124. Adenoviruses encoding human IL‐10 or beta‐galactosidase significantly prolong the survival time of LT grafts by maintaining hepatocellular integrity and liver function 125. Furthermore, injection of adenoviruses encoding human IL‐10 conserves hepatic integrity by suppressing the NFκB signalling pathway and increasing the expression levels of HO‐1 and Bcl‐2 126. The potential regulated mechanism is attributed to diminished activities of cytochrome c and caspase‐3 and simultaneous up‐regulation of HO‐1 and Bcl‐2 expression 125.

Under the development of operative technology, an increasing number of patients with end‐stage‐liver disease have received LT to prolong the survival time, and most of the grafts for clinical use are steatotic or cadaveric. Additionally, few detailed investigations of the molecular signalling pathways in human LT models exist to allow the study of regulatory mechanisms.

## Hepatic IR exerts various effects according to liver health

Although pre‐treatments *in vivo* and *in vitro* significantly maintain mitochondrial function and liver function, liver tissue responds to hepatic IR differently and exhibits extremely different reactions of ROS generation according to the health of liver 127. Steatotic and aged liver models are much more sensitive to external stimulations 128, 129. Fatty livers exhibit decreased MMP and delayed repolarization 113, and these models are particularly vulnerable to hepatic IR 130. Additionally, the unhealthy state of steatotic livers increases the level of large lipid droplets and ROS generation but decreases ATP‐dependent energy metabolism 129. There is an abundance of cholesterol and depolarization in the hepatic mitochondria of steatotic livers; thus, the beneficial mitochondrial GSH level is significantly decreased 131.

Mitochondrial function in fatty livers significantly decreases after 5 hrs of cold preservation, because the energy metabolism of steatotic livers is impaired, and the primary dysfunction is increased compared with that in lean livers^132^. Chu *et al*. 133 have demonstrated that steatotic livers exhibit significant mitochondrial dysfunction by altering the activity of mitochondrial complex I, and are more susceptible to prolonged cold ischaemia in LT models. Consequently, steatotic livers are excluded for partial liver transplantation according to the baseline health of the liver 134. Because steatotic and aged liver organs are easily impaired in function, the regulatory pathways help to improve the physiologic and morphologic outcomes of hepatic tissues.

Although steatotic livers are highly sensitive to injury, some pre‐conditional protocols are also effective in improving mitochondrial function and liver function after hepatic IR injury. Although IPC exerts no effect on mitochondrial function, it significantly normalizes the function of fatty livers 135. The protective effect of suppressing mitochondrial MPT in pre‐conditional protocols is more significant in young rats than in old rats after hepatic IR injury 128. Metformin pre‐conditioning attenuates the hepatic IR‐induced necro‐inflammatory reaction and mitochondrial dysfunction, decreases the levels of serum aminotransferases, and eliminates lipoperoxidation in high‐fat diet‐fed rats 136. Three inhibitors of autophagy including 3‐methyladenine, bafilomycin A1 and exendin 4 protect against hepatic IR injury by decreasing the expression levels of autophagy‐associated proteins and maintaining mitochondrial function in obese mice 130. Tauroursodeoxycholic acid decreases the hepatic inflammatory response after partial hepatectomy by inactivating mitochondrial anion channels, decreasing the release of cytochrome c and activating the level of caspase‐9 signalling; consequently, the apoptosis and necrosis rates of hepatocytes are decreased and liver regeneration is promoted in steatotic livers 3. Although APC has been found to be un‐protective in an early stage of hepatic IR in a steatotic mouse model, it has been proven to be an effective protective agent in a late stage through the adenosine monophosphate kinase signalling pathway 137. It is important to investigate protocols to eliminate lipid droplets and reverse the fat deposition in liver tissue, thereby allowing effectively the maintenance of liver function after warm or cold IR and increasing the survival rate in various pathological conditions.

## Conclusions

Pathological states can be categorized into various categories including liver with underlying diseases, hepatic partial hepatectomy, trauma, haemorrhagic shock, cardiac arrest, LT state. Mitochondrial dysfunction is an important cellular event contributing to hepatic IR, and the restoration of mitochondrial function sustains cell and tissue survival *in vivo* and *in vitro* models. All ischaemia and reperfusion processes trigger a series of inflammatory responses including the activation of tissue macrophages and recruitment of neutrophils. Although mitochondrial dysfunction may be immediately initiated, and ROS formation causes higher rates of cell damage, the regulation of mitochondrial function maintains the balance of energy metabolism and normal hepatic function. Because of the mechanisms between warm hepatic IR and cold hepatic IR are not the same, treatments should be investigated according to its own features for potential clinical therapies.

More strategies are under investigation and are being mechanistically tested to improve the proliferation rate or decrease the death rate of hepatocytes or other non‐parenchymal cells in a hepatic IR injury state. As our review demonstrated, various mechanisms participate in the pathological process of hepatic IR, the mechanisms vary according to different experimental conditions including *in vitro* or *in vivo* condition, type of ischaemia, period of ischaemia, graft subclinical situation, etc. Therapeutic interventions including IPC, pre‐treatments with non‐physiological oxygen content, pharmaceutical pre‐conditioning and genetic modifications may be promising strategies to improve the antioxidant capacity in hepatic IR models *in vitro* and *in vivo*. Since gene modification shows vector toxicity and low transfection efficiencies, and leads to adequate mutants in warm or cold hepatic IR models. In addition, siRNA and micro RNA targetting specific genes to elaborate release of inflammatory and cell death factors is an advanced approach for hepatic IR. Because of complex procedures including partial or whole liver procurements, conservation in destined buffer, organ rewarming and final transplantation in LT models, the isolated liver tissues cumulatively acquire irreversible injuries. Pathological processes should be inhibited by effective routes which exert less side‐effect on human bodies. LT is an emergency procedure and leaves very little time to pre‐treat the donor with genetic approaches, thus it may be impossible to exert gene modification on clinical patients to improve the outcome of hepatic IR. Furthermore, some drugs mentioned above have possible side‐effects and frequently limit their use in human LT procedure. Moreover, elimination of LT‐induced injury should be focused on the donor organ's health, patient's own health and the precise surgical procedures. Although some procedures in our review may be unrealistic for clinical usage, these procedures and their regulative pathways help to clarifying the underling mechanisms for hepatic IR. Then, clinical therapies can be effectively developed for improving long‐term survival rate during various pathological processes.

## Conflicts of interest

The authors declare no conflicts of interest.
